# Modeling the crosstalk between malignant B cells and their microenvironment in B-cell lymphomas: challenges and opportunities

**DOI:** 10.3389/fimmu.2023.1288110

**Published:** 2023-11-02

**Authors:** Baptiste Brauge, Elise Dessauge, Florent Creusat, Karin Tarte

**Affiliations:** ^1^ UMR 1236, Univ Rennes, INSERM, Etablissement Français du Sang Bretagne, Equipe Labellisée Ligue, Rennes, France; ^2^ SITI Laboratory, Centre Hospitalier Universitaire (CHU) Rennes, Etablissement Français du sang, Univ Rennes, Rennes, France

**Keywords:** tumor microenvironment, follicular lymphoma, diffuse large B-cell lymphoma, germinal center, stromal cells, 3D models, xenografts, genetically-engineered mouse models

## Abstract

B-cell lymphomas are a group of heterogeneous neoplasms resulting from the clonal expansion of mature B cells arrested at various stages of differentiation. Specifically, two lymphoma subtypes arise from germinal centers (GCs), namely follicular lymphoma (FL) and GC B-cell diffuse large B-cell lymphoma (GCB-DLBCL). In addition to recent advances in describing the genetic landscape of FL and GCB-DLBCL, tumor microenvironment (TME) has progressively emerged as a central determinant of early lymphomagenesis, subclonal evolution, and late progression/transformation. The lymphoma-supportive niche integrates a dynamic and coordinated network of immune and stromal cells defining microarchitecture and mechanical constraints and regulating tumor cell migration, survival, proliferation, and immune escape. Several questions are still unsolved regarding the interplay between lymphoma B cells and their TME, including the mechanisms supporting these bidirectional interactions, the impact of the kinetic and spatial heterogeneity of the tumor niche on B-cell heterogeneity, and how individual genetic alterations can trigger both B-cell intrinsic and B-cell extrinsic signals driving the reprogramming of non-malignant cells. Finally, it is not clear whether these interactions might promote resistance to treatment or, conversely, offer valuable therapeutic opportunities. A major challenge in addressing these questions is the lack of relevant models integrating tumor cells with specific genetic hits, non-malignant cells with adequate functional properties and organization, extracellular matrix, and biomechanical forces. We propose here an overview of the 3D *in vitro* models, xenograft approaches, and genetically-engineered mouse models recently developed to study GC B-cell lymphomas with a specific focus on the pros and cons of each strategy in understanding B-cell lymphomagenesis and evaluating new therapeutic strategies.

## 1 Introduction

The germinal center (GC) reaction is the finely controlled process allowing the selection and differentiation of high-affinity B cells (1). In secondary lymphoid organs (SLOs), cognate antigen exposure triggers B-cell activation and migration to the T:B border where CD4^pos^ T cell help allows them to seed early GC. Within GC, dark zone (DZ) centroblasts proliferate and undergo random somatic hypermutations in the variable region of Immunoglobulin (Ig) genes. Mutant GC B cells then migrate to the light zone (LZ), where only high-affinity centrocytes receive survival and activation signals from cognate GC-residing T follicular helper cells (Tfh), allowing them to recirculate to the DZ for additional cycles of proliferation and mutations, and to ultimately differentiate into memory B cells and plasma cells. Most B cells are not positively selected and undergo apoptosis before being cleared by tingible-body macrophages. Several lymphoid stromal cell (LSC) subsets have been described that support each step of B cell recruitment, activation, survival, and differentiation within SLOs (2, 3). Follicular dendritic cells (FDCs) specifically organize the LZ/DZ segregation within GC. DZ-FDCs produce CXCL12, which attracts CXCR4^hi^ proliferating centroblasts. LZ-FDCs produce CXCL13, which recruits CXCR5-expressing centrocytes and Tfh, and retain intact antigens for extended periods, thus providing the substrate for affinity-dependent selection. Furthermore, several non-GC LSC subsets with specific localizations and functions, including T-cell zone reticular cells, marginal reticular cells, interfollicular reticular cells, T-B border reticular cells, and medullary reticular cells, collectively referred as fibroblastic reticular cells (FRCs), contribute to mature B-cell differentiation by recruiting naïve B and T cells, transferring antigens to B cells, organizing activated B and T cell trafficking and interactions within LN, or mediating plasmablast and plasma cell migration and survival. As a whole, normal B-cell activation within SLOs is strongly dependent on discrete microanatomic areas supported by specialized LSC subsets and controlling B-cell fate through direct B-cell supportive functions and through the recruitment and polarization of other immune cell subsets.

Mature B-cell neoplasms are a group of heterogeneous diseases corresponding to clonal expansion of mature B cells arrested at various stages of differentiation and disseminated preferentially within lymph nodes (LNs) and bone marrow (BM) (4). Among them, follicular lymphoma (FL) (5) and diffuse large B-cell lymphoma (DLBCL) (6) are the most frequent lymphomas arising from GC/early post-GC B cells, but they are characterized by opposite patterns of tumor microenvironment (TME) composition and organization (7). FL is an indolent lymphoid malignancy but about 20% of patients experience early progression and poor outcome. Additionally, nearly 30% of FL cases can transform into aggressive lymphoma (tFL) (8). FL B cells retain key features of normal GC B cells, such as a macroscopic organization in well-defined follicles without DZ/LZ segregation and a strong dependency of a GC-like TME both within invaded LNs and within FL-infiltrated BM (2, 9). FL-infiltrating immune and stromal cells are engaged in a dynamic, bidirectional crosstalk with FL B cells. Specific genetic hits have an impact on the development of distinct TME profiles (5) and, in turn, the fine-tuned balance between pro-tumoral and anti-tumoral signals supports FL B-cell heterogeneity and subclonal evolution within permissive niches. In agreement, the majority of predictive biomarkers in FL are related to TME features. By contrast, DLBCL tumors are densely packed with tumor B cells and have been proposed as less dependent on surrounding non-malignant cells (10). In accordance, the most popular prognostic classification of DLBCL tumors, *i.e*. GC B cell (GCB)- *versus* activated B cell (ABC)-DLBCL, relies on tumor cell genetic characteristics reminiscent of their cell of origin and molecular transformation pathways. However, recent studies have identified a landscape of TME ecosystems capturing DLBCL clinical heterogeneity beyond genotypic classifications (11) and have highlighted a key role for DLBCL-infiltrating stromal cells (12, 13).

Several questions are still unsolved concerning the crosstalk between lymphoma B cells and their microenvironment, including the impact of the kinetic and spatial heterogeneity of tumor niche on B-cell heterogeneity, and how individual genetic alterations trigger B-cell intrinsic but also B-cell extrinsic signals driving the reprogramming of non-malignant cells. The clinical relevance of B-cell/TME interplay is also a matter of debate, with potential impacts on patient stratification, response to treatment, and design of therapeutic strategies. The major pitfall to answer these questions is the paucity of relevant models integrating tumor cells harboring specific genetic hits, non-malignant immune and stromal cells, extracellular matrix (ECM), and biomechanical forces. Here, we review the different tools used to study B-cell lymphomas, interrogating the pros and cons of each technique, and their potential to better understand tumor heterogeneity and develop novel therapeutic options.

## 2 *In vitro* models of B-cell lymphomas

Historically, most *in vitro* studies investigating B-cell lymphoma biology and drug response used suspension cultures of lymphoma cell lines, disregarding the lymphoma niches. Recently, new models have been introduced that incorporate TME cell subsets or TME-derived signals and/or move from standard 2D cultures to 3D systems including ECM components and biomechanical forces (**Table 1**).

**Table 1:** Pros and Cons on *in vitro* lymphoma models.

Dimension	Model	Tools	Pros	Cons
**2D**	Monoculture	Tumor B cell lines or primary lymphoma cells	Simple and cheap Reproducible Fully automatable	Limited predictivity of drug efficiency in patients No TME
	Coculture	Tumor cells and stromal cells +/- T cell-dependent signals	Partly reproduced tumor niche Cell-cell interactions	No spatial organization No biomechanical forces
**3D**	MALC	Lymphoma cell lines Hanging drop method Ultra-low attachment plates	Simple and cheap High throughput (amenable for drug screening) Possibility of image analysis	Lack of ECM Lack of TME No primary lymphoma B cells
	Static natural hydrogel-based coculture	Lymphoma B cells + Col1/Hystem/alginate + stromal cells +/- T-cell dependent signals	Useful to test drugs with B-cell intrinsic and extrinsic activities Possibility to add numerous TME components Biomechanical properties Tested with primary DLBCL	Batch-to-batch variability Limited stability Limited design flexibility
	Static synthetic hydrogel-based coculture	Lymphoma cell lines + Functionalized PEG-MAL + stromal cells +/- T-cell dependent signals	Versatile Controlled ECM architecture and functional properties	More complex to set up No primary lymphoma cells
	Dynamic synthetic hydrogel-based coculture	Lymphoma cell lines + Functionalized PEG-MAL + stromal cells Microfluidic system	Control of mechanical constraints Evaluation of shear stress effect	Expensive and complex set up Low throughput No primary lymphoma cells
	Encapsulation in alginate spheroids	Lymphoma B cells + Matrigel + stromal cells Alginate capsule	External and internal constraints High throughput Permeable, Versatile Tested with primary FL	No shear stress Decreased drug diffusion

DLBCL, Diffuse Large B-cell Lymphoma; ECM, extracellular matrix; FL, Follicular Lymphoma; MALC, Multicellular aggregates of lymphoma cells; PEG-MAL, maleimide functionalized polyethylene glycol; TME, Tumor microenvironment.

### 2.1 2D models

Cell lines are the easier way to study tumor cells, owing to their homogeneity in culture and capacity to be modified on purpose. In the context of B-cell lymphomas, a large panel of DLBCL cell lines are available, and have been extensively characterized for their genetic, transcriptomic, and drug response profiles, recapitulating the main features of either ABC- or GCB-DLBCL. In contrast, established FL B cell lines only correspond to tFL, requiring the use of primary FL B cells for the study of FL biology. However, despite their overexpression of the anti-apoptotic factor Bcl2 through the t(14;18) translocation, FL B cells do not survive *in vitro* in the absence of a supportive TME. The protumoral TME components that have been widely used to maintain FL B cell survival and proliferation *in vitro* are CD40L and IL-4, mimicking the interaction with FL-Tfh (14–16), which overexpress both factors within invaded LN. More recently, CD40L has also been shown to be efficient in sustaining the survival and drug resistance of ABC-DLBCL *in vitro (* 17). In addition to Tfh, stromal cells are another key component of lymphoma TME frequently used in *in vitro* models (18). Stromal cells obtained from both SLOs (and called Resto or HK cells) or BM support *in vitro* the survival of primary FL B cells, in particular after priming with tumor necrosis factor-α (TNF) and lymphotoxin-α1ß2 (LT), that trigger their commitment towards functional LSC-like cells (19–22). Adipose-derived stromal cells could also be used as LSC precursors to improve FL and DLBCL B cell viability *in vitro (* 23, 24). Of note, these reductionist models are unable to consider LSC heterogeneity and plasticity while FL-LSCs and DLBCL-LSCs exhibit specific transcriptomic and functional profiles (13, 25). In addition, mature FDCs could not be maintained or differentiated from stromal precursors *in vitro* and could thus not be evaluated in this context. Interestingly, the combination of IL-4 with TNF/LT upregulates VCAM-1 and CXCL12 in stromal cells *in vitro* and *in vivo*, thus stimulating FL B cell migration and activation. These data demonstrate the interest of combining Tfh-derived signals with stromal cells to mimic FL TME (23). Altogether, these 2D models are still widely used, due to their simplicity, cost-effectiveness, reproducibility, and uniform distribution of stimuli, drugs, and cell interactions. However, as reported in solid cancers, growing evidence shows that spatial organization and biomechanical forces are significant determinants of lymphoma niches and pushes the development of 3D models integrating these parameters.

### 2.2 3D models

The most classical 3D model is based on the hanging drop method, which enables cells to create multicellular tumor spheroids. In the context of lymphoma, multicellular aggregates of lymphoma cells (MALC) have been used to demonstrate that the transcriptomic profile of tumor B cell lines grown in 3D conditions is closer to that of primary FL B cells than classical 2D suspension cultures (26). This tool was subsequently used to evaluate different therapeutic approaches, such as chemotherapy agents, anti-PD1 antibodies, and natural- and antibody-dependent cell cytotoxicity in coculture with NK or Tγδ cells (26–29). However, the hanging drop method is inadequate for drug screening due to the manual transfer of MALC into agarose-precoated wells. A more recent adaptation of this method was proposed where MALC were induced to self-aggregate in ultra-low attachment plates, enabling analysis of drug response through 3D imaging technologies and specific image processing pipeline (30). However, a major limitation of MALC-related approaches remains the absence of ECM architecture and TME cell components.

To address these concerns, alternative methods have been developed based on natural or synthetic hydrogel scaffolds that create ECM-like biophysical properties and allow for the addition of TME cell subsets (31). The crosstalk between B-cell lymphoma and BM stromal cells has been studied in agarose hydrogel spheroids using a DLBCL cell line and HS-5 cell line as a surrogate of BM mesenchymal stromal cells (32). The presence of stromal cells was shown to reduce drug-induced apoptosis in malignant B cells. In an attempt to incorporate immune cell components to lymphoma *in vitro* models, type-I collagen was used as a naturally-derived ECM to create 3D spheroids of DLBCL cell lines with TNF/LT/IL-4-treated adipose-derived stromal cells and monocyte-derived macrophages, creating a platform to test antibody-dependent cell phagocytosis (24). Similarly, a mixture of alginate, a natural polymer devoid of cell-binding properties, with puramatrix, a synthetic peptide promoting cell adhesion, was used in a high-throughput microfluidic-based platform to create lymphoma spheroids consisting of a GCB-DLBCL cell line, HS5 stromal cell line, and allogenic peripheral blood mononuclear cells (33). These spheroids were found useful to test the effects of Lenalidomide, a drug with tumor-intrinsic and tumor-extrinsic activities in DLBCL. Finally, the HyStem-C hydrogel was used in a mouse lymphoma-on-chip model where the hydrogel was embedded within a solid polydimethylsiloxane macrostructure and traversed by a microchannel perfused with endothelial cells, recapitulating tumor microvascularization (34). This model allowed for the association of various primary immune murine cells, thus mimicking lymphoma TME. The use of engineered stromal cells co-expressing CD40L and B-cell activating factor was proven interesting to recapitulate normal B-cell lymphoid microenvironment (35) and would be interesting to test for lymphoma B cells.

Natural ECM-based models have some technical limitations such as batch-to-batch variability, limited thermal and mechanical stability, uncontrolled enzymatic degradation, and limited design flexibility. In contrast, maleimide functionalized polyethylene glycol (PEG-MAL) offers several advantages, including tunable functionalization with integrin-binding adhesive peptides and crosslinking using protease-degradable thiol-containing crosslinkers enabling matrix degradability (36). These versatile synthetic hydrogel organoids support a specific role for α4ß1 integrin signaling (triggered by the VCAM-1-mimicking ‘REDV’ peptide) above αvß3 integrin signaling (triggered by the fibronectin-mimicking ‘RGD’ peptide) to promote proliferation of the HBL-1 DLBCL cell line in the presence of SLO-derived stromal cells (37). These 3D organoids, including ECM and stromal cells, upregulate BCR expression and reduce drug-induced B-cell apoptosis. Likewise, CD40L presentation in PEG-MAL functionalized with REVD induces cooperative signals in DLBCL, including the BCR and TLR pathways, which result in resistance to MALT1 and kinase inhibitors (17).

LNs are dynamic structures expanding and becoming mechanically stiff under immune cell recruitment and proliferation, while the resolution of the immune response is associated with LN contraction and a return to a baseline of mechanical softness (38). Neoplastic LNs are chronically expanded with a stiffness about two-fold greater than that in non-malignant LNs (39), suggesting a major role for biomechanical forces in lymphoma physiopathology. By adjusting the hydrogel crosslinking density, the fitness of functionalized PEG-MAL organoids increases, which in turn, increases CD40L-dependent Src kinase phosphorylation in ABC-DLBCL cell lines (17). Furthermore, a microfluidic bioreactor was designed to investigate the impact of low fluid shear stress, reported earlier in the subcapsular sinus lumen of lymphoid tissues, on DLBCL cell growth (40). Fluid flow induces an upregulation of BCR and integrin receptors on ABC-DLBCL cell lines, together with an increased cell proliferation and a decreased drug-induced apoptosis. These data definitively demonstrate that microenvironment-mediated biophysical forces are crucial for B-cell lymphomagenesis, even though the microfluidic model used here remains difficult to handle and low-throughput.

LN mechanical properties depend on both external constraints induced by the capsule and internal constraints created by the dense networks of LSCs that produce and contract ECM. To account for these different parameters, we developed a 3D model based on high-throughput generation of size-controlled, permeable, and elastic alginate shells. In this model, lymphoma B cell lines are encapsulated together with SLO-derived stromal cells and/or a layer of ECM lining the inner wall of the capsules (41). In contrast to classical 3D culture approaches that embed cells in an ECM scaffold, B cells and stromal cells are engaged in a self-organization process in alginate spheroids, where stromal cells are anchored and spread onto ECM, thus forming a ramified 3D network that supports B cell proliferation. Stromal cells variably increase tumor growth and drug resistance in spheroids depending on the lymphoma subtype and the therapeutic compound tested. Finally, while only primary DLBCL cells have been successfully maintained in other 3D models (17, 24), the co-culture in 3D alginate microspheres supports the survival of primary FL B cells.

Overall, *in vitro* 3D models represent a valuable tool to explore the crosstalk of lymphoma B cells with their physical and cellular microenvironment, due to their reduced cost, reproducibility, and versatility. By combining large panels of B-cell lines, that can be genetically edited on purpose, defined ECM scaffolds, and selected non-malignant immune and stromal cell subsets, 3D models offer a unique opportunity to explore the mechanisms underlying bidirectional cell interactions within the lymphoma niche, and screen new therapeutic approaches before proceeding to *in vivo* models. However, given the extremely complex organization of the lymphoma TME, the inability to purify/maintain/differentiate *in vitro* functional stromal cell subsets, and the need for autologous LN immune cells to reproduce their specific tumor-infiltrating features and avoid the bias of allogenic reactivity, several gaps remain unfilled, generally requiring a combination of *in vitro* and *in vivo* models.

## 3 *In vivo* models of B-cell lymphoma transplantation

**Figure 1:**
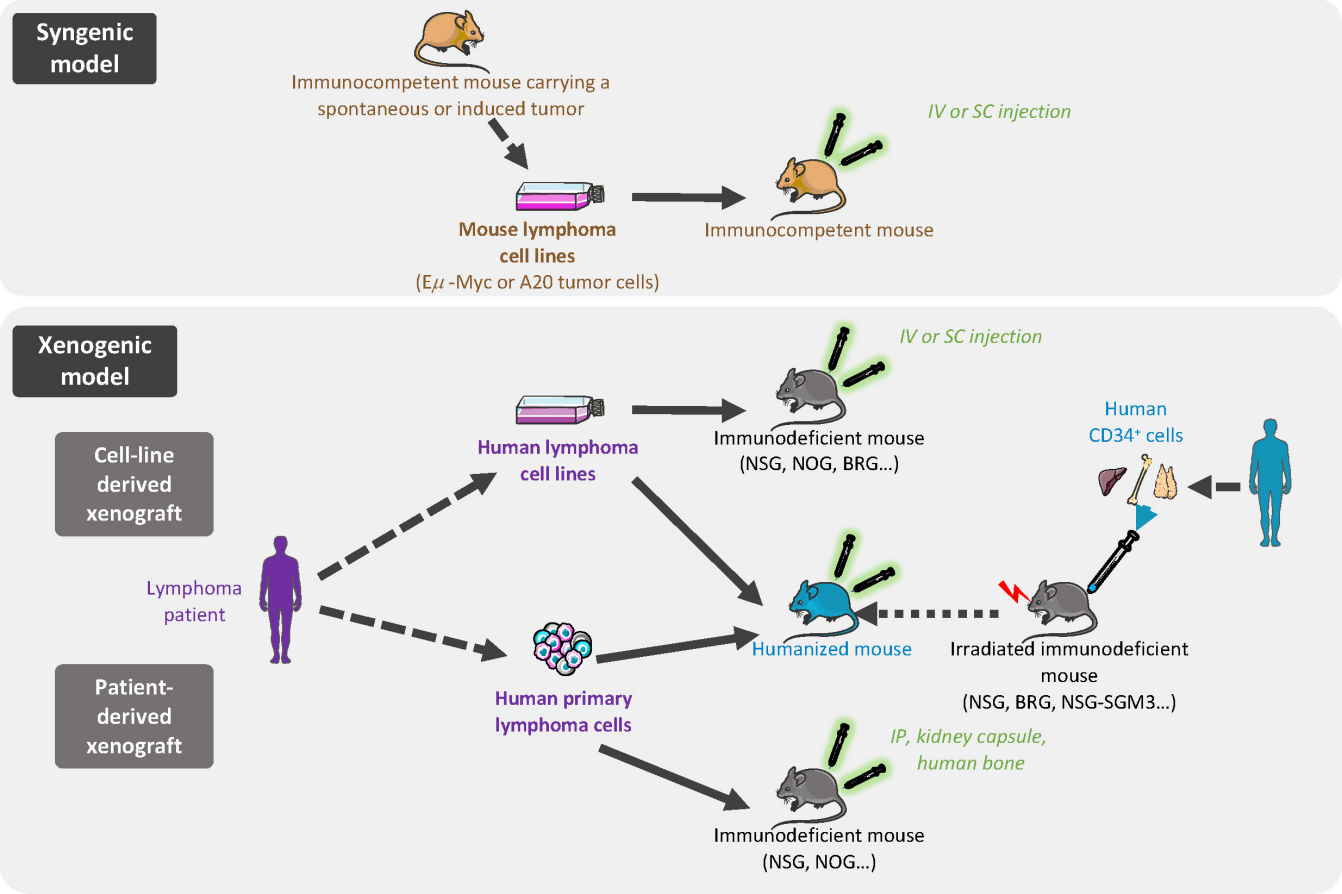
*In vivo* models of B-cell lymphoma transplantation. In syngenic models, murine cells are derived from immortalized mouse lymphoma cells from the same inbred strain as the immunocompetent recipient mice and are implanted by intravenous (iv) or subcutaneous (sc) injection. Xenogeneic models are based on human lymphoma cell line (cell-line derived xenograft) or primary lymphoma cells (patient-derived xenograft) grafted in immunocompromised or humanized mice.

The ideal model for exploring cancer biology and novel therapies involves tumor cells recapitulating the spectrum of primary human cancer cell characteristics, growing within the appropriate local TME, in a context of functional immune system. Additionally, this ideal model should be fast and not too expensive. Although it is clearly impossible to achieve all of these conditions, a wide range of *in vivo* models have been developed, notably based on tumor cell transplantation, allowing various issues to be addressed (**Figure 1**).

### 3.1 Syngenic mouse models

With the aim of evaluating tumor growth in a context of a functional immune system and TME, syngenic models enable the study of mouse tumors in a fully competent system. These easy, low cost, and reproducible models offer the possibility to investigate the inter-relationship between TME and lymphoma cancer cells, and to evaluate the efficacy of immunotherapy approaches (**Table 2**). In syngenic models, the transplanted cells are immortalized mouse cancer cells from the same inbred strain as the recipient mice thus avoiding the need for immunosuppressive regimens.

**Table 2:** Pros and Cons of *in vivo* models of lymphoma transplantation.

	Pros	Cons
**SYNGENIC MODELS**	Low cost, fast, high-yield, and reproducible Fully competent murine system permitting to evaluate the efficacy of immunotherapy and the role of TME on lymphoma progression Possibility to compare various administration routes Possibility to genetically edit tumor cells before implantation	Difficulty to predict the success of human therapy by evaluation of the equivalent therapy in this murine system Lack of lymphoma murine cell lines fully mimicking their human counterpart The widely-used subcutaneous route implicates a lack of lymphoid TME Inability to study the initiation of lymphoma and early stages of tumorigenesis
**XENOGRAFT MODELS** **Cell lines xenograft in immunodeficient mice**	Low cost, fast, high-yield, and reproducible Panel of tumor cell lines mimicking primary tumor features Possibility to genetically edit tumor cells before implantation Possibility to compare various administration routes	Lack of immune TME Inability to study the initiation of lymphoma and early stages of tumorigenesis
**XENOGRAFT MODELS** **Patient-derived xenograft in immunodeficient mice**	Excellent representation of primary lymphoma characteristics, retention of human tumor heterogeneity Relevant to study therapeutic options on human lymphoma cells, and to investigate personalized drug therapy	Need for some technical skills Low engraftment rate of primary B-cell lymphomas Progressive loss of human TME Inability to study the initiation of lymphoma and early stages of tumorigenesis
**XENOGRAFT MODELS** **Cell lines or patient-derived xenograft in humanized mice**	Competent human immune system to study immune response role on lymphoma growth and drug response	Need for some technical skills Expensive and time-consuming

Such approaches have been used to characterize lymphoma/TME crosstalk. As an example, intravenous grafts of cells from tumor-bearing *Eμ-Myc* mice were used to interrogate the interplay between lymphoma B cells and LSCs and the remodeling of LN endothelial cells by tumor B cells (42, 43). Interestingly, these transplantable models allow a control of the kinetics of the tumor. In addition, the subcutaneous infusion of A20 mouse lymphoma B cells is widely used to study anti-lymphoma therapies. Recently, treatment of A20-transplanted mice with the hypomethylation agent azacytidine revealed that tumor cell DNA methylation pattern contributes to TME features (11). Moreover, the same model has shown the benefit of combining immunotherapy strategies to overcome immune escape in advanced B-cell lymphomas (44–46). In the past decade, chimeric antigen receptor (CAR) T cells have emerged as a treatment of choice for relapse/refractory B-cell lymphoma. The evaluation of the interactions between CAR-T cells, tumor cells, and TME could be done in immunocompetent syngenic mouse models. Specifically, transplantation of *Eμ-Myc* tumor cells was used to describe the critical role of CAR-T cell/TME crosstalk in promoting host T cell and NK cell activation, stimulating CAR-T cytotoxic activity, and ultimately inducing tumor regression (47). Finally, transplanted cells can be genetically modified prior to implantation. The impact of tumor burden on rituximab exposure and efficacy was explored using a syngenic lymphoma cell line expressing human CD20 and the luciferase enzyme (48). Moreover, B-cell lines were modified to express a caspase 3 reporter and a calcium sensitive dye in order to precisely evaluate the functional consequence of cytotoxic T cell activity. This revealed that the interactions between lymphoma B cells and host cytotoxic T cells or CAR-T cells are mostly unproductive or sublethal (49). Tumor cells and host recipients can also be humanized for therapeutic targets through genetic engineering. For example, huCD47-A20 syngenic tumor cells were injected into a quadruple knocked-in huPD1xhuPD-L1xhuCD47xhuSIRPα immunocompetent mouse to evaluate a bispecific antibody designed to target PD1 and CD47 (50).

Despite the advantages of these allograft mouse tumor systems, they show significant limitations for the study of FL/DLBCL. First, murine cell lines often fail to accurately mimic human lymphoma B cells and several differences between human and mice lymphomas have to be considered, such as clinical features and genetic characteristics, often showing different driver mutations. Although widely used, the *Eμ-Myc* transgenic model cannot be considered as a DLBCL model. Lymphomas from *Eμ-Myc* mice are heterogeneous and range from the pre-B to the mature B-cell stages, with a majority of pre-B/immature B-cell lymphomas, in agreement with the window of activity of the Eμ Ig promoter (51). At the mature stage, *Eμ-Myc* lymphomas generally correspond more closely to Burkitt-like lymphomas. A20-derived tumors are closer to DLBCL with an immune desert-TME and do not represent the broad spectrum of ecosystems described in this disease (11). Moreover, no transplantable murine FL model has been described so far. Importantly, most of the studies used intravenous or subcutaneous injections that are not associated with an initial homing into SLOs. Whether intrasplenic or BM infusion would modify tumor engraftment and TME evolution remain to be explored.

### 3.2 Human xenograft mouse models

Complementary studies using human xenograft models are often required to confirm the relevance of the data generated using *in vitro* or syngenic mouse models and remain the gold-standard for preclinical research, being relatively simple, inexpensive, high-yield, and reproducible (**Table 2**). These models are based on subcutaneous infusion, often combined with a Matrigel scaffold, or intravenous infusion of human lymphoma cell lines in immunocompromised mice. In this context, a broad spectrum of DLBCL/tFL cell lines, representing all genetic subtypes, can be used to predict the subset of patients concerned by the pathological pathways or the new therapeutic approaches being tested. Subcutaneous xenograft models were used to investigate the oncogenic roles of BCL6/NOTCH2, BCL2, or EZH2 and to evaluate corresponding specific inhibitors, thus paving the way for their further evaluation as therapeutic agents (52–55). Intravenous infusion enables the spontaneous colonization of various tissues including SLOs, thus mimicking the broad dissemination profile of most lymphomas and opening the possibility to compare tumor growth across different niches.

For all xenograft models, immunodeficiency of mice is essential to prevent human cell rejection and ranges from nude athymic mice (nu/nu), deficient in mature circulating T cells, to many variations of the historical model of severe combined immunodeficiency mice (SCID) (56). Mutations in *prkdc* (SCID mice) or *Rag* (RAG mice) genes abrogate mouse adaptive immune response but are not sufficient for proper engraftment of lymphoid malignancies, as these mice retain partial innate immunity. Moreover, the SCID mouse model has limited usage due to the development of mouse B and T cells upon aging. To achieve maximum tumor engraftment, mice increasingly immunocompromised are used. Mutations in the interleukin 2 receptor common gamma chain (IL2rγ), an important component of cytokine signaling, lead to severe dysfunctions of the innate immunity, including an absence of murine NK cells. Three strains of IL2rγ-deficient mice, especially receptive to tumor graft, are widely used: NSG/NOG mice (NOD.*Prkdc^scid^.IL2r*γ*^null^* mice, harboring respectively a complete deletion *versus* a truncation of the intracellular signaling domain of IL2rγ) and BALB/c.*Rag2^null^.IL2r*γ*^null^* (BRG) mice. Interestingly, the NOD background is more supportive of human cells due to a specific mutation in *Sirpa* conferring a higher sensitivity to the human don’t-eat-me receptor CD47, resulting in resistance of human cells to phagocytosis by murine macrophages (57).

The primary drawback of using these models is the inability to study the impact of immune response on tumor growth. To overcome this limitation, humanized mice co-engrafted with human tumors and a human immune system have emerged as a suitable strategy, particularly for evaluating immunotherapy approaches (58). The conventional strategy to create humanized mice is to engraft CD34^pos^ human hematopoietic stem and progenitor cells (HSPCs), obtained from human fetal liver, umbilical cord blood, or BM, into irradiated NSG or BRG mice. Once transplanted, HSPCs establish in the BM and differentiate into T and B lymphocytes, macrophages, and other human cells capable of interacting with grafted tumor cells. Several parameters affect the engraftment of HSPCs and the quality of immune regeneration, such as the background and age of the recipient, the HSPC source and injection route, and the preconditioning regimen. As such, this approach is complex, expensive, and time-consuming. In addition, classical humanized mouse models exhibit dysfunctions of innate and adaptive immunity, related to the lack of human cytokines and a limited development of LN structure related to a deficiency in Il2rγ-dependent lymphoid tissue inducer cells. Improving the cytokine environment of humanized mice is associated with a better development of human innate immune cells. NSG mice expressing human stem cell factor, GM-CSF and IL-3 (NSG-SGM3) display increased numbers of human B cells and myeloid cells after HSPC transplantation and have been used to study the adverse effects of CD19-targeting CAR-T cells. Specifically, CAR-T infusion in NSG-SGM3 mice has uncovered a role for recipient macrophages in cytokine-release syndrome (59), which is the most frequent complication of CAR-T cells in leukemia and lymphoma patients. Furthermore, a new strategy to overcome the lack of LN development was recently proposed, based on the expression of mouse thymic stromal lymphopoietin in BRG mice deficient for *Sirpa* (BRGS), leading to an improved development of LN, thymic structure, and Tfh, associated with the generation of antigen-specific antibodies (60). Given the key role of Tfh and LN microenvironment in the development of FL, it is tempting to speculate that such improvement of mouse humanization will open the possibility to engraft primary FL B cells. Yet, no mouse model allows the efficient engraftment of primary FL or DLBCL B cells into recipient LNs.

### 3.3 Patient-derived xenograft models

The use of patient-derived xenografts (PDX) is a major step forward to better recapitulate primary lymphoma characteristics, including heterogeneity, morphology, molecular features, and interaction with TME (**Table 2**). Although a large number of PDX models have been developed for solid tumors, there is limited data available in hematological diseases. Corresponding to transplant of lymphoma tumors intraperitoneally (61), into an implanted human bone chip (62), or under the kidney capsule (63), lymphoma PDX avoid *ex vivo* culture responsible for genotypic and phenotypic changes in cancer cell lines. DLBCL PDX have been demonstrated to retain the histology, mutational profile, copy number variants, and drug sensitivity of the primary tumors (63). They have therefore been proposed as a suitable platform for drug screening. Importantly, human TME progressively disappears from the lymphoma graft, especially after serial transplantation (63), hindering the study of B-cell/TME interactions. Nonetheless, in the context of very aggressive double-hit DLBCL, rearranged for both MYC and BCL2, PDX models have brought out the crucial role for BM macrophages in mediating the clinical effect of alkylating agents, suggesting compartment-specific mechanisms of resistance. In line with the strong dependency of FL B cells on immune and stromal TME, very few PDX models of FL have been published (62, 64) and they have been poorly characterized. Interestingly, a new approach has recently emerged to derive short-term FL PDX in avian embryos allowing to capture clinical heterogeneity in response to immune-chemotherapy (65). To sum up, the various lymphoma graft models have significant restrictions when it comes to study tumor cell/TME crosstalk: i) murine B-cell lymphomas fail in truly mimicking the molecular and cellular heterogeneity of their human counterparts; ii) despite humanization, the immunocompromised hosts used for lymphoma xenografts exhibit a variably defective TME; iii) PDX essentially lose their human TME; and iv) the engraftment rate of primary FL B cells remain extremely low. In addition, whatever the model, a major limitation is the inability to study the initial steps of lymphomagenesis and the kinetics of TME reprogramming. Genetically engineered mouse models (GEMM) represent complementary models providing a great benefit to the understanding of the mechanisms of tumor initiation and evolution.

## 4 Genetically-engineered lymphoma mouse models

**Figure 2:**
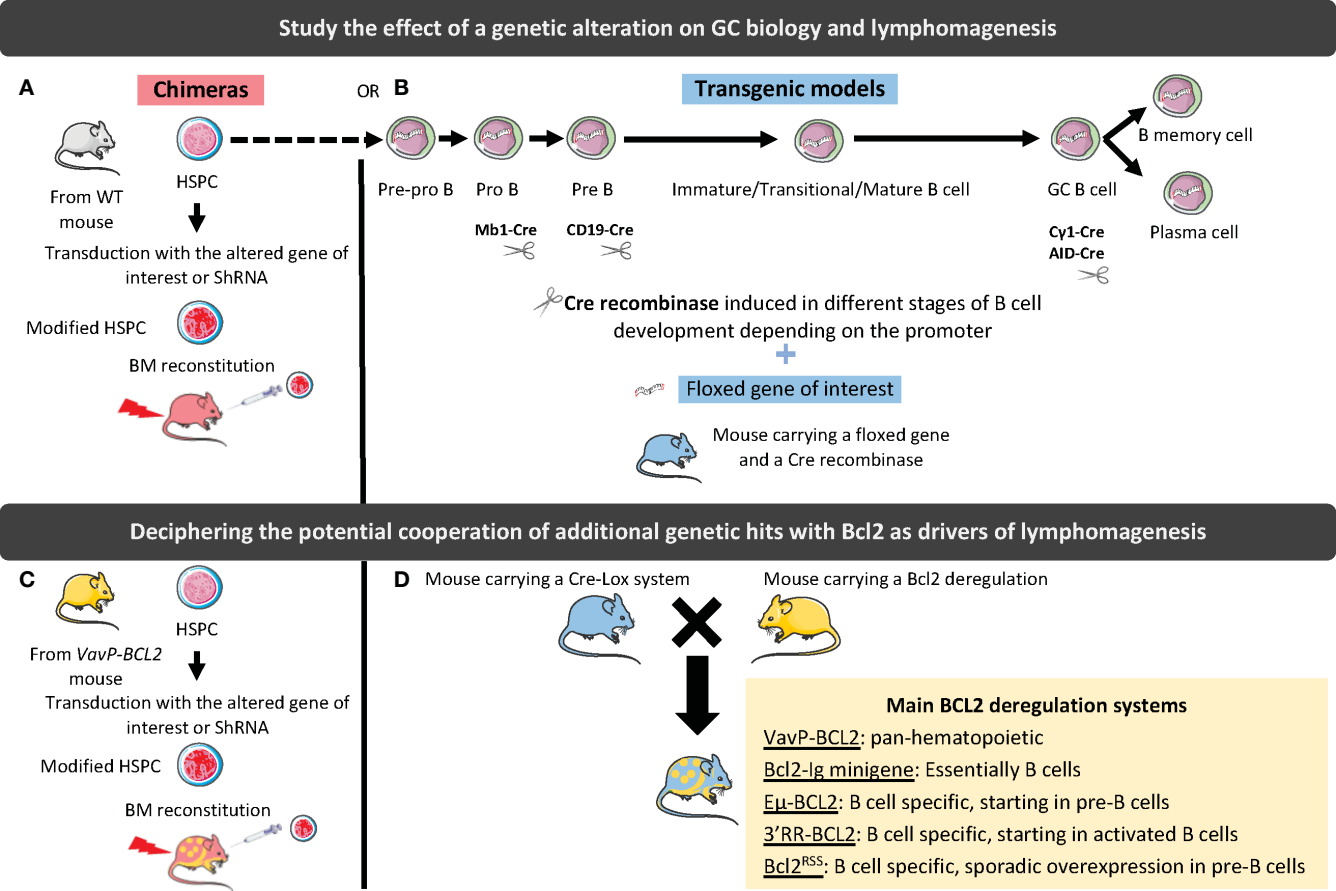
Strategies to study the effect of genetic alterations on GC biology and lymphomagenesis. In chimeric mouse models, hematopoietic stem and progenitor cells (HSPCs) are harvested from WT **(A)** or BCL2-deregulated **(B)** mouse and transduced with a vector driving the silencing (shRNA), or the overexpression (WT or mutated gene) of the gene of interest. Transduced cells are then used to reconstitute WT mouse BM. In transgenic mouse models, a CRE recombinase under the dependence of a promoter deregulating the gene of interest at a specific stage of B-cell differentiation is associated with a floxed gene of interest **(C)**. These models can be crossed with BCL2-deregulated mouse models **(D)**. The different tools to deregulate BCL2 are indicated in the yellow box.

The molecular and cellular complexity along with the spatial and kinetic heterogeneity of mature B-cell lymphomas have pushed the development of GEMM making it possible to: i) gain insights into normal GC biology and regulation; ii) study the contribution of individual recurrent genetic hits alone or in combination to lymphomagenesis and drug sensitivity; iii) decipher the co-evolution of tumor B cells and TME from early pre-lymphoma steps to overt lymphoma and tumor progression; iv) assess the effects of therapeutic agents in relevant lymphoma models. In addition to the nature of the selected genetic hits, the design of GEMM must account for several parameters (**Figure 2**). The first parameter to consider is the type of model, which can either be based on homogeneous transgenic mouse genotypes that can be crossed to generate more complex models, or on BM chimera where HSPCs are transduced with a vector coding for a mutated gene or for a shRNA targeting this gene before they are transferred into irradiated hosts. Generating and crossing GEMM is a long and complex process but it results in homogeneous cohorts of mice while BM chimera is more rapid to generate but produce a limited number of heterogeneous chimeric recipients. The second important parameter is the method used to induce genetic hits, that is generally i) conditional using the Cre-Lox system, targeting either the entire B-cell compartment (CD19-Cre, Mb1-Cre) or the GC B cells (Cγ1-Cre, AID-Cre), and ii) inducible under tamoxifen treatment. Finally, the tools used to characterize GEMM, *i.e.* histology, flow cytometry, transcriptomic analysis (including scRNAseq), evaluation of B-cell clonality, as well as the kinetics of analysis, *i.e*. prelymphoma versus overt lymphoma stages, are highly variable. Overall, very heterogeneous GEMM have been generated making it difficult to compare the effects of each individual genetic alteration.

Among the numerous lymphoma models recently published, those mimicking aggressive ABC-DLBCL, deregulating Myd88 or BTG1 (66, 67), have not been studied for their TME and will not be further developed in this review. Conversely, GC-lymphoma models, including FL and GCB-DLBCL, were shown to display important remodeling of the GC niche that support the initial stage of lymphomagenesis. They are essentially based on the deregulation of the anti-apoptotic gene *BCL2*, mimicking the founder t(14;18) translocation common to the pathogenesis of FL and GCB-DLBCL, and a second hit affecting GC B cell differentiation (68).

### 4.1 BCL2 deregulation

The BCL2/IGH translocation, occurring during V(D)J rearrangement in the BM, is considered the first transformative event in the pathogenesis of FL (5, 69). However, it is not enough to transform B cells, as evidenced by the identification of t(14;18)^pos^ post-GC memory-like IgM^pos^ B cells in the peripheral blood of a majority of healthy individuals (70). Due to their resistance to apoptosis, translocated B cells survive the GC reaction and undergo iterative entry into GC, accumulating additional genetic hits ultimately leading to overt FL.

Various strategies have been developed to model this founder t(14;18) translocation. In particular the *VavP-BCL2* transgenic mice, where human BCL2 is controlled by the pan-hematopoietic regulatory sequence *VavP*, have been shown to develop a FL-like disease with a cumulative incidence of about 40% at 18 months of age (71). Of note, 20% of mice developed early fatal autoimmune kidney disease, suggesting a role for autoreactivity in this model. The massive GC hyperplasia seen in *VavP-BCL2* mice is highly dependent on CD4^pos^ T cell help. This model reproduces thus some of the main features of indolent FL and has been widely used thereafter as a backbone for additional genetic hits. However, the amplification of CD4^pos^ T cells was shown to rely on their strong overexpression of BCL2, underlying the need of a mouse model where the deregulation of BCL2 is specific to B cells.

The first B-cell specific model of BCL2 deregulation was based on a human *BCL2-Ig* minigene mimicking the t(14;18) translocation and resulted in the development of polyclonal lymphoid hyperplasia involving B cells and plasma cells and to an increased probability of developing GC-experienced lymphomas, including FL-like and plasmablastic-like lymphoma (72, 73). These data demonstrate that overexpression of BCL2 in B cells is not associated with a block of B cell differentiation at the GC stage. Similarly, the *Eµ-BCL2* transgenic strain, which places human BCL2 under the control of the 5’ *IGH* enhancer E*µ*, shows an expansion of B cells and plasma cells, associated with autoimmune kidney disease but without increased incidence of lymphoma (74). In mature B cells, the main locus driving Ig expression is not the 5’ E*µ* promoter but the IgH locus 3’ regulatory region (3’RR) (75). Another model was thus developed in which the murine 3’ enhancer region was inserted into the murine *Bcl2* locus, resulting in massive Bcl2 overexpression restricted to B cells, an impairment of B-cell lymphopoiesis, and a development of FL-like GC tumors within 7 to 14 months (76). In this context, T-cell homeostasis was not altered but the mice displayed a polyclonal plasmacytosis suggesting that FL development relies on late secondary hits arising on a subset of B cells and triggering an accumulation at the GC stage. Unfortunately, this model was not further characterized. More recently, another attempt to model FL-like *BCL2* deregulation has been proposed (77). In this study, human *BCL2* was introduced either by knock-in at the *Igκ* locus, resulting in a pan-B cell deregulation, or as a transgene under the control of the *3’RR* enhancer, resulting in induction of BCL2 expression in early GC B cells. Upon iterative sheep red blood cell (SRBC) stimulation, *Igκ-BCL2* resulted in massive accumulation of plasmablasts and long-lived plasma cells, while *3’RR-BCL2* lead to an accumulation of GC B cells, further highlighting the key role of the mechanism of BCL2 deregulation. Ultimately, both genotypes developed plasmablastic lymphoma, confirming that BCL2 overexpression does not restrain GC B-cell differentiation. Finally, a human *BCL2^RSS^* mouse model has been engineered to mimic the t(14;18) translocation in only a few B cells at the pre-B cell stage through the introduction of RAG recombination sites at the vicinity of an inactivated *BCL2* minilocus (78). This model, reproducing the low frequency of pre-FL/cancer precursor cells found in healthy individuals, was used to confirm the hypothesis of the iterative GC transit of cancer precursor cells, thereby triggering AID-mediated genomic instability. Again, BCL2-expressing B cells do not progress to overt FL.

Collectively, these different models have confirmed that BCL2 deregulation is crucial but not sufficient to trigger early FL/GCB-DLBCL pathogenesis. Furthermore, they have revealed that the mechanism of BCL2 deregulation may influence lymphoma development, but also TME features and interactions with B cells, suggesting it should be carefully selected to design relevant models.

### 4.2 Epigenetic alterations

**Figure 3:**
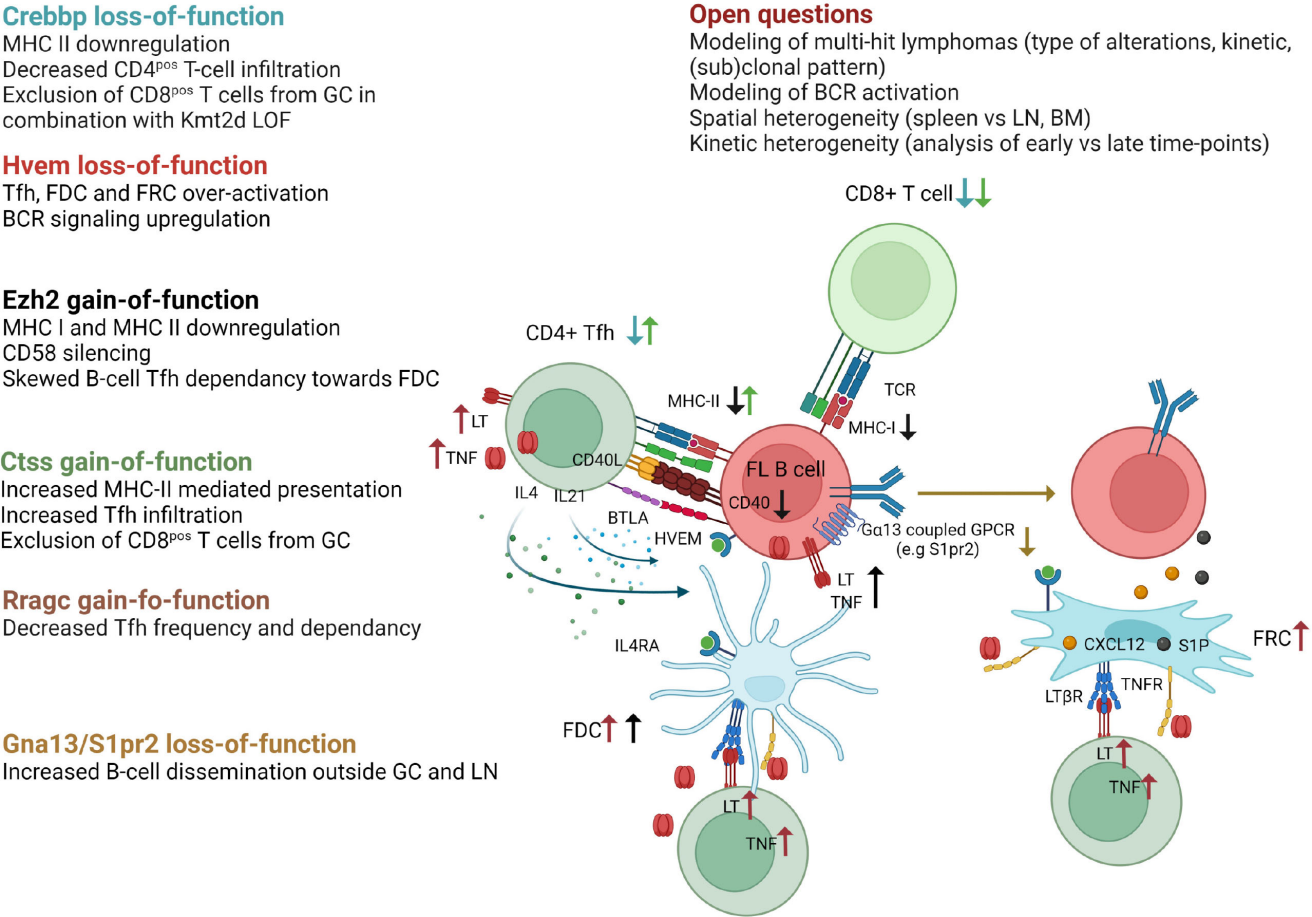
Impact of FL recurrent genetic hits on the crosstalk between B cells and their TME as revealed in GEMM. *Crebbp* LOF is associated to a loss of MHC class II expression preventing antigen presentation to CD4^pos^ T cells as well as a decreased infiltration of CD4^pos^ and CD8^pos^ T cells within TME. When combined with *Kmt2d* loss-of-function, *Crebbp* alteration leads to an exclusion of CD8^pos^ T cells from GCs. *Hvem* loss-of-function triggers over-activation of Tfh, FDC, and FRC, and upregulates BCR signaling. *Ezh2* gain-of-function mutations skew the Tfh dependence of tumor B cells towards FDC, in part because such mutations yield CD40, MHC-I, and MHC-II downregulation, and leads to CD58 silencing. *Ctss* GOF mutations favor an increase in Tfh infiltration and MHC-II presentation, while preventing CD8^pos^ T cells to infiltrate the tumors. *Rragc* activating mutations lead to resistance to nutrient withdrawal and decrease the need for Tfh support. Finally, loss-of-function of the confinement receptors *Gna13* or *S1pr2* is associated with B-cell dissemination outside GC and LN. The main remaining questions in the field are indicated. Generated with Biorender.

Recurrent somatic mutations in histone/chromatin modifying enzymes are a hallmark of GC-derived lymphomas and are collectively found in nearly 100% of FL and the vast majority of GCB-DLBCL. A large panel of GEMM interrogating lymphoma epigenetic alterations is now available as recently reviewed (68). Interestingly, the 3 most common alterations target CREBBP, KMT2D, and EZH2 and, in addition to their B-cell intrinsic activity, have all been shown to impact on the capacity of tumor B cells to interact with, and eventually modify, their TME (**Figure 3**).

Loss-of-function (LOF) mutations of the lysine acetyltransferase CREBBP are very early events in FL pathogenesis, already detectable in cancer precursor cells (79), while LOF mutations of the lysine transferase KMT2D are the most common epigenetic hits in this disease (80). Conditional deletion of *Crebpp* or *Kmt2d* in mouse B cells results in GC hyperplasia and increases the incidence of GC lymphoma in BCL2-driven mouse models after iterative SRBC immunization, with a spectrum of histopathological features ranging from early FL to DLBCL (80–84). Of note, the deletion of *Crebbp* in B cells is not sufficient *per se* to induce oncogenic transformation (85). Conversely, *Kmt2d* deficiency was variably associated with a lack of cancer development or with the development of non-GC aggressive lymphomas, depending on inactivation strategy, *i.e. Cγ1^cre^Kmt2d^fl/fl^* mice (81) or *CD19^cre^Kmt2d^fl/fl^* mice (80), respectively. These data clearly confirm the cooperation between BCL2 deregulation and CREBBP/KMT2D LOF in GC lymphoma and, again, the importance of the model design, including the timing and mechanism of genetic alterations. Mechanistically, CREBBP counteracts the repressive effects of BCL6 through H3K27 acetylation at enhancers of BCL6 target genes, leading to GC exit. CREBBP mutations abrogate the capacity of GC B cells to respond to exit external stimuli thereby promoting lymphoma development (82–84). Similarly, the transcriptional program affected by *Kmt2d* loss is enriched in GC LZ-related genes, including genes associated with BCR/CD40 signaling (80, 81). In addition, GEMM studies have revealed a specific role for *Kmt2d* in class-switching. Notably, LOF mutations of CREBBP and KMT2D co-occur in about 50% of FL and 30% of a subset of GCB-DLBCL even though they regulate overlapping pathways (86). Combining *Crebbp* and *Kmt2d* haploinsufficiency under the control of *Cγ1-Cre* identified a direct physical interaction of Crebbp and Kmt2d on enhancers critical for GC LZ reaction and a direct acetylation of Kmt2d by Crebbp, thereby modulating Kmt2d activity (86). Combined *Crebbp/Kmt2d* defect synergizes *in vivo* to trigger abnormal GC expansion and accelerates the onset of FL-like B-cell lymphoma after crossing with *VavP-BCL2* mice. Interestingly, *CREBBP* mutations in human FL/DLBCL, as well as in murine lymphomas, have been associated with a huge reduction in MHC class II expression, together with a defective antigen presentation and a decreased CD4^pos^ T cell infiltration, showing how a single genetic hit can affect the composition of the lymphoma niche (87, 88). Furthermore, combined inactivation of *CREBBP/KMT2D* was shown to specifically trigger CD8^pos^ T cell exclusion from malignant follicles in both human and murine lymphomas (89). Recently, *CREBBP* LOF has also been proposed to increase the expression of CSF1 and CCL2 in DLBCL tumor cells, thereby promoting tumor-associated macrophage recruitment and polarization towards immunosuppressive macrophages (90). However, this phenomenon has not been reported yet in GEMM, although it has been described in DLBCL PDX. Altogether, CREBBP and KMT2D alterations emerged as key drivers of GC B-lymphoma, displaying synergistic effects on B-cell transformation and impacting immune escape and niche reprogramming.

Heterozygous gain-of-function (GOF) mutations of the EZH2 methyltransferase, in most cases affecting an evolutionarily conserved residue (Y641), are found in about 30% of FL and GCB-DLBCL. EZH2 contributes to normal GC formation by repressing the CDKN1A cell-cycle checkpoint and silencing plasma cell gene expression (91, 92). GC B cell-specific knock-in of mutant *Ezh2* carrying the Y641 activating mutation (*Cγ1^cre^ Ezh2^Y641F/wt^*) results in GC hyperplasia, whereas deletion of *Ezh2* or Ezh2 inhibitors abrogate GC formation and Ig affinity maturation (91, 93). *Ezh2* overactivation at the GC B-cell stage is not sufficient for lymphoma development, but earlier activation (*CD19^cre^ Ezh2^Y641F/wt^*) could lead to aggressive lymphomas (94). *Ezh2* GOF mutation cooperates with BCL2 deregulation (*VavP-BCL2, Cγ1^cre^ Ezh2^Y641F/wt^*) to increase the frequency of lymphomas with features of GCB-DLBCL (93, 95). Again, in addition to these B-cell intrinsic activities, *EZH2* activating mutations have been shown to contribute to B-cell crosstalk with TME. First, they are associated with MHC class I and MHC class II loss in mouse models and human lymphomas, and pharmacological inhibition of EZH2 enhances MHC class I expression in GCB-DLBCL cell lines (95, 96). *EZH2* GOF mutations also induce CD58 epigenetic silencing in lymphoma cells, associated with a decreased NK cell activation, suggesting that lymphoma B cells harboring *EZH2* mutations can escape both T cell and NK cell lysis (97). Finally, competitive BM chimeras revealed that *Ezh2* GOF mutation is associated with an alteration of the capacity of premalignant GC B cells to interact with Tfh and stromal cells (98). In particular, mutant GC B cells are less dependent on Tfh help while accumulating in an FDC-dependent manner as LT-overexpressing CC. These data were confirmed in FL patients, where the FDC network was found to be more expanded in EZH2 mutant patients.

CREBBP, KMT2D, and EZH2 are thus frequent epigenetic hits, initially considered as direct tumor inducers regulating B-cell proliferation and differentiation, but now widely recognized for their additional role as modulators of lymphoma TME, in particular based on the data obtained in GEMM. Other alterations of epigenetic regulators, including linker histones (H1) (99), MEF2B (73) or SETD2 (100), are regularly found in FL/GCB-DLBCL, but their putative impact on TME composition and crosstalk with tumor B cells has not yet been interrogated in corresponding GEMM.

### 4.3 Non-epigenetic lymphoma drivers

Over the past 5 years, GEMM targeting non-epigenetic targets have provided a lot of crucial data to unravel normal and malignant GC biology, revealing how B cells interact with their surrounding microenvironment (**Figure 3**). In particular, the role of the sphingosine 1-phosphate receptor 2 (S1PR2) in GC B-cell confinement was initially identified in mouse models where its inactivation was sufficient to trigger the development of clonal GC hyperplasia and GCB-DLBCL in aged mice (101, 102). These data revealed that S1PR2 is upregulated in normal GC B cells and acts through Gα13 to dampen Akt activation and inhibit cell migration to the S1P^hi^CXCL12^hi^ outer follicle thus maintaining B cells in the follicle centers. Interestingly, GCB-DLBCL frequently exhibit deleterious mutations in *S1PR2*, but also in *GNA13* (the gene coding for Gα13), or in the Gα13 effector *ARHGEF (* 103). Association of *G*α*13* deletion in all B cells (Mb1^cre^Gα13^fl/fl^ mice) and early Bcl2 deregulation (Eµ-BCL2 mice) in mixed BM chimera cooperate in promoting GC B cell survival and dissemination outside the GC niche with a frequent BM involvement. The lack of such a huge dissemination profile in *S1pr2* KO mice, leads to the identification of another Gα13-coupled inhibitory receptor, P2RY8 (103), with redundant activities on GC B cell confinement through interaction with *S*-geranylgeranyl-L-glutathione (104), and recurrent mutations in GCB-DLBCL. Of note, no murine homolog of P2RY8 was identified. Altogether, these data shed new light on the critical role of the disruption of GC B cell confinement in GC B-cell lymphomagenesis. Interestingly, BM involvement is more frequent in FL than in DLBCL patients without reported mutations in Gα13 migration pathway. However, S1PR2 expression was found to be reduced in FL B cells compared to normal CC (25), suggesting additional levels of regulation for this crucial pathway and confirming the difficulty to mimic FL disease in GEMM.

FL-like disease was obtained in mice deregulating BCL2 and HVEM/TNFRSF14, one of the most frequently inactivated genes in FL (40% of cases). Deletion of *Hvem* in hematopoietic stem and progenitor cells of VavP-BCL2 mice followed by infusion into irradiated WT mice accelerated GC B cell expansion and the development of tumors with phenotypic and histologic features of FL (105). Interestingly, these lymphoma-prone mouse models also revealed for the first time the dual role of HVEM in normal GC biology. First, Hvem interaction with its inhibitory ligand Btla in cis restrains BCR-dependent B cell activation, identifying a cell-autonomous effect for *Hvem* loss. In addition, Tfh are known to express high levels of BTLA. HVEM-BTLA loop dampens Tfh activation and resulting B-cell help. In agreement, *Hvem*-deficient mice exhibit amplification of Tfh producing Il4 and Il21, but also increased amount of Tnf/Lt associated with an overactivation of FDC and FRC. *Btla* deficiency in T cells similarly leads to GC expansion and accelerates lymphomagenesis (106) in a B cell-extrinsic manner. These data translate to human, since *in vitro* stimulation of human Tfh with soluble HVEM specifically increases their production of TNF/LT, while FL patients harboring HVEM mutations have higher numbers of Tfh within infiltrated follicles. Furthermore, they open up an interesting therapeutic opportunity by exploiting the capacity of engineered CAR-T cells to deliver *in situ* anti-tumoral soluble HVEM (105).

Two other genetic alterations affecting Tfh infiltration were found to be mutually exclusive with HVEM LOF in FL patients: GOF alterations of Cathepsin S (*CTSS*) and LOF alterations of Ras-related GTP-binding protein (*RRAGC*). Cathepsin S has critical functions in antigen presentation on MHC class II molecules and thus in communication with CD4^pos^ T cells. CTSS activation is found in about 20% of FL patients either through GOF mutations or through gene amplification (107). *VavP-BCL2* HSPCs transduced with human *CTSS* prior to infusion into irradiated WT mice leads to a rapid development of FL-like tumors characterized by an accumulation of Tfh, while CD8^pos^ T cells are excluded from the GC (108). Consistently, FL patients with deregulated *CTSS* have a higher intrafollicular CD4^pos^ T-cell infiltration, supporting a stronger dependence on Tfh help and a key role of CTSS mutations in TME co-opting in FL. Conversely, point-activating mutations in the mTORC1 activator *RRAGC* are associated with a decrease in Tfh abundance and an accelerated development of FL-like tumors in *VavP-BCL2*; RagC^mut^ mice and FL patients harboring *RRAGC* mutations (18% of FL cases) (109, 110). In this context, *Raggc* mutations are associated with a cell-intrinsic selective survival advantage, making GC B cells less dependent on Tfh cell help but highly sensitive to the mTOR inhibitor rapamycin. Of note, inactivation of *SESTRIN1* by deletion or by its epigenetic silencing by *EZH2* GOF mutations also favors mTORC1 activation and synergizes with *VavP-BCL2* to induce FL-like tumors but in this context the modifications of the TME have not been studied (111).

Whether other recurrent genetic hits in FL, including silencing mutations of EPHA7, or alterations in the retinoblastoma proliferation pathway, might affect the way tumor B cells interact with their niche has not been evaluated in the relevant GEMM (112, 113).

The development and detailed characterization of GEMM have been instrumental in our current understanding of the bidirectional crosstalk between GC B cells and their niche, and how specific genetic hits can directly or indirectly alter these interactions to promote lymphomagenesis. However, several major questions remain unanswered. First, with the exception of the combination of *Crebbp* and *Kmt2d* LOF (86), no GEMM has been developed to evaluate the co-occurrence of multiple genetic hits with BCL2 upregulation, although FL and GCB-DLBCL are characterized by numerous genetic alterations. Whether such combined alterations should occur in the same clone or in different subclones co-existing within the tumor, and what would be the best sequential timing for each additional hit, is not clearly defined and deserves new relevant models. Second, no GEMM integrates the role of BCR signaling in FL pathogenesis. FL is characterized in about 95% of cases by the introduction of N-glysosylation sites within the variable regions of Ig, which are occupied by immature oligomannose residues allowing BCR crosslinking by DC-SIGN-expressing tumor-associated macrophages (114, 115). Such weak and long-lasting BCR activation is difficult to reproduce in mice in the absence of a DC-SIGN ortholog. Conversely, all murine lymphoma models are driven by repeated immunization by SRBC, which induces a very strong GC reaction not reflecting human lymphomagenesis. Third, murine lymphoma models develop tumors essentially in the spleen, whereas FL/GCB-DLBCL develop within LN and eventually BM, where immune and stromal cells have a different composition and organization. Fourth, the timing of analysis is crucial, and very few studies offer, in the same model, a characterization of the impact of genetic hits on i) early pre-tumoral B cells/B-cell niche; ii) established FL-like tumors; iii) transformed lymphomas. Such work would require large cohorts of homogeneous GEMM sacrificed at different time points. Finally, the age of mice was not fully taken into account in the different lymphoma mouse models, whereas microenvironment ageing can influence tumor progression and response to therapy. In particular, an accumulation of senescent fibroblasts, a decrease of ECM integrity, an increase in systemic low-grade chronic inflammation or an increase expression of immune checkpoint have been described in aged mice (116).

## 5 Conclusion

Given the rising importance of the TME in B-cell lymphomagenesis and drug response, there is a growing need for relevant and reproducible models interrogating the dynamic crosstalk between tumor cells and their surrounding niche, while recognizing the inherent limitations of the various models used.

Recently, 3D *in vitro* systems have acquired greater complexity, allowing the integration of TME cell subsets, microvascularization, and biomechanical forces. Thus, with the advantage of enabling custom genetic editing of the tumor B cells and being potentially high-throughput, these systems are useful to ask some specific questions about lymphoma/TME crosstalk mechanisms or to design drug screening approaches. *In vivo* models of B-cell lymphoma transplantation are very diverse, with the possibility of working with a full immune microenvironment in the case of syngenic transplants, or with tumor B cells as relevant as possible to human GC B-cell lymphomas, in the case of xenografts. Humanized mice and PDX provide additional refinement, but also complexity and cost, which limit their use for initial large-scale strategies. Finally, GEMMs have contributed significantly to our understanding of the interplay between normal and malignant GC B cells and their niche by recapitulating the natural history of lymphoma development and evolution. However, they need to reach a higher level of complexity, as a single genetic hit is not sufficient to mimic the complex genetic landscape of human lymphomas. Ultimately, all these models should hopefully serve to transfer more rapidly and efficiently new therapeutic strategies into the clinic.

## Author contributions

BB: Writing – original draft, Writing – review & editing. ED: Writing – original draft, Writing – review & editing. FC: Writing – original draft, Writing – review & editing. KT: Conceptualization, Funding acquisition, Writing – original draft, Writing – review & editing.
